# Phytotoxicity of zinc oxide nanoparticles and multi-walled carbon nanotubes, alone or in combination, on *Arabidopsis thaliana* and their mutual effects on oxidative homeostasis

**DOI:** 10.1371/journal.pone.0281756

**Published:** 2023-02-15

**Authors:** Shaohui Yang, Rong Yin, Chen Wang, Yongkui Yang, Jiehua Wang

**Affiliations:** School of Environmental Science and Engineering, Tianjin University, Nankai Area, Tianjin, China; St Cloud State University, UNITED STATES

## Abstract

The extensive use of engineered nanoparticles (ENPs) has raised concerns about their potentially harmful effects on the ecosystem. Despite previous reports of a variety of individual ENPs, the mutual effects of ENPs when used in combination were not well understood. In this study, we first investigated the effects of different sizes and concentrations of ZnO nanoparticles (ZnO NPs) or multi-walled carbon nanotubes (MWCNTs) on the growth performance of *Arabidopsis thaliana* seedlings. Then, two concentrations of ZnO NP (40 and 50 mg/L) with a diameter of 90 nm and MWCNTs (100 and 500 mg/L) with an outer diameter of 40–60 nm were used to evaluate their respective or simultaneous phytotoxicity to Arabidopsis. The results showed that seedlings exposed to either ZnO NPs or MWCNTs exhibited significant phytotoxic symptoms. ZnO NPs caused stronger inhibitory effects than MWCNTs on several plant growth indices, including reduced root length, chlorophyll content, and increased ROS concentration. When applied together, the concurrent effects of ZnO NPs and MWCNTs on Arabidopsis seedlings appeared to be more negative, as evidenced not only by the further deterioration of several growth indices but also by their synergistic or additive regulation of the activities of several antioxidant enzymes such as superoxide dismutase (SOD), catalase (CAT) and glutathione reductase (GR). Moreover, qRT-PCR analysis revealed that in the presence of ZnO NPs and MWCNTs, the expression of genes important for maintaining cellular ROS homeostasis was differentially regulated in shoots and roots of Arabidopsis seedlings. Overall, our data may provide new insights into how plants respond to more than one type of nanomaterial and help us better understand the associated environmental risks.

## Introduction

Nanoparticles (NPs) are receiving increasing attention due to their nano size (1–100 nm) and unique physicochemical properties, including their small size and high surface-to-volume ratio [1]. NPs are manufactured and used in a variety of commercial products, and their inevitable release into soil, water, and atmosphere after use [2, 3] has raised significant concerns about their potential negative impact on the environment [4]. Since plants are the largest interface between the environment and the biosphere, they play a critical role in the fate and transport of nanoparticles in the environment [5–8]. Therefore, it is particularly important to understand the interactions between plants and nanoparticles.

Zinc oxide nanoparticles (ZnO NPs) are widely used for their photolytic properties, and there is growing concern about their fate and toxicity in the environment [9]. The toxicity of ZnO NPs to many plants has already been reported for *Arabidopsis thaliana* [10, 11], *Fagopyrum esculentum* [12], *Triticum aestivum* [13], *Spirodela punctuta* [14] and *Cucumis sativus* [15], *Medicago sativa* [16]. In Arabidopsis, for example, not only did high concentrations of ZnO NPs (400, 2000, 4000 mg/L) show toxic effects on seed germination, root elongation, and number of leaves [10]. In addition, Nair et al. reported that treatment with 20 mg/L ZnO NPs caused changes in root morphology and treatment with 50 mg/L reduced plant fresh weight and primary root length [11]. The 50% inhibitory concentration (IC_50_) of ZnO NPs is about 50 mg/L for radish and about 20 mg/L for ryegrass and canola [17]. It has been shown that the mechanism underlying the phytotoxicity of ZnO NPs is its ability to cause oxidative stress and promote the formation of reactive oxygen species (ROS) [14, 16]. Meanwhile, plants develop a variety of mechanisms involving enzymatic antioxidants such as superoxide dismutase (SOD), peroxidase (POD), ascorbate peroxidase (APX), and catalase (CAT), as well as non-enzymatic substances such as glutathione (GSH) and ascorbic acid (AsA), to scavenge ROS and reduce the toxic effects of NPs [8, 18]. However, due to the diversity of NP types and plant species, it is difficult to infer a general antioxidant defense response in plants to different NP exposures [19].

Carbon nanotubes (CNTs) have unique physical and chemical properties. They are being actively explored for applications in computing, aerospace, biomedical, and other industries [20]. However, there are few reports on the toxicity of multi-walled carbon nanotubes (MWCNTs) and the interaction between MWCNTs and plants in vitro and in vivo. MWCNTs showed a negative effect on the growth of rice cells due to the increase of reactive oxygen species (ROS) and caused a decrease in cell viability and density [21]. Moreover, agglomerates of MWCNTs were toxic to Arabidopsis suspension cells by decreasing the dry weight, viability, chlorophyll content, and SOD activity of the cells [22]. Compared to other NPs, single-walled carbon nanotubes (SCNTs) have the unique ability to easily penetrate the cell wall and cell membranes [23]. In Arabidopsis mesophyll protoplasts, a low dose of SCNTs stimulated cell growth, but a high concentration caused necrosis, apoptosis, and the formation of ROS [24] as a dose-dependent two-phase control. Compared to unexposed seedlings, soybean seedlings treated with 100 μg/mL MWCNTs increased total fresh shoot weight [25], but 20 mg/L MWCNTs inhibited soybean growth and decreased dry biomass weight [26]. Exposure of MWCNTs at a concentration of 10–40 mg/L could also stimulate seed germination and promote tomato seedling growth [27] by affecting the expression of genes essential for cell division and plant development [28]. Tomato plants grown in soil enriched with MWCNTs produced two times more flowers and fruits compared to control plants [29]. However, MWCNT suspensions at a concentration of 2 g/L showed no significant effect on root growth of radish, canola, ryegrass, lettuce, corn, and cucumber compared to the control [16]. Moreover, the presence of MWCNTs in rice suspension cell cultures increased the ROS level and decreased cell viability [21].

The recent study focused on the co-contamination between CNTs and a pharmaceutical compound in a model agricultural soil [30, 31]. A study on four agricultural crops showed that multi-walled CNTs reduced the accumulation of chlordane and DDx, DDT and its metabolites, in the soil in a dose-dependent manner [30]. This indicates that CNTs in the environment can significantly affect the bioavailability and translocation pattern of coexisting organic pollutants [31]. Moreover, ZnO-MWCNTs nanocomposites have been widely used in recent years due to their high thermal conductivity [32], strong photocatalytic activity [33], good sensitivity and fast response to UV radiation [34]. While the literature consistently highlights the toxicity of individual NPs, there are few data on the combined effects of multiple NPs on plant growth. In the present study, we first investigated the phytotoxic effects of ZnO NPs and MWCNTs at different sizes and concentrations on Arabidopsis thaliana seedlings. Then, we selected two concentrations for each nanomaterial at a specific size to investigate their mutual effects when applied simultaneously. The data obtained in this study should provide new insights into how plants respond to more than one type of nanomaterial and help us better understand the associated environmental risks.

## Materials and methods

### Nanoparticle treatment of Arabidopsis seedlings

Zinc oxide nanoparticles (ZnO NPs) and the multi-walled carbon nanotubes (MWCNTs) were purchased from Macklin Biochemical Co, Ltd (Shanghai, China) and Shenzhen Nanogang Co, Ltd (Shenzhen, China), respectively. Information on the materials provided by the manufacturer can be found in S1 Table. The nanomaterials were dispersed in ultrapure water by ultrasonic treatment (ultrasonic homogenizer JY92-IIN, 100W, 20-25KHz) for 1 h to allow homogeneous suspension and reduce the aggregation of ZnO NPs and MWCNTs in their stock solutions. Seeds of wild-type Arabidopsis (ecotype Col-0) were sterilized with 75% ethanol for 5 minutes and 1% sodium hypochlorite (V/V) for 10 minutes before vernalization at 4°C for 3 days. Subsequently, half-strength Murashige and Skoog (MS) medium [35] was supplemented with ZnO NPs of diameter (90 and 200 nm) at concentrations (15, 30, and 50 mg/L) [11] and MWCNTs of outer diameter (10–20, 20–40, and 40–60 nm) at concentrations (50, 100, 200, and 500 mg/L) [24]. The media were solidified immediately by keeping the vessels at 4°C to avoid aggregation and precipitation of NPs ½ MS agar medium without NPs was used as a control.

Seeds were grown on solid ½ MS medium (pH 5.7) containing 1% (w/v) sucrose, 0.8% (w/v) phytoagar, and various sizes and concentrations of nanomaterials. Petri dishes were then placed vertically in a growth chamber under a constant 16h/8h (day/night) regime at an ambient temperature of 22°C/18°C and a light intensity of 9600 lx. Plant samples were collected after an exposure period of 10 days for morphological, physiological and gene expression analyses.

### Morphological and chlorophyll content analyses

Arabidopsis seedlings were photographed, and the length of primary roots and hypocotyls was measured using the Image J program (http://rsb.info.nih.gov/ij). Approximately 0.2 g of fresh Arabidopsis leaves were homogenized with 95% ethanol (v/v) to extract chlorophyll as described previously [36]. The absorbance of the supernatant at 649 and 665 nm wavelengths was determined using a spectrophotometer, and the chlorophyll a (Chl-a), chlorophyll b (Chl-b), and total chlorophyll contents were calculated as previously described [37].

### Analysis of ROS contents, enzymatic and non-enzymatic antioxidants activities

Fresh roots and shoots of Arabidopsis seedlings were homogenized in liquid nitrogen to a fine powder and vigorously extracted for 5 minutes in ice-cold 50 mM phosphate buffer (pH 7.0) containing 0.1% Triton X-100 and 1% polyvinylpyrrolidone PVP-40 (w/v). The mixture was then centrifuged (Heittch MIKR022R) for 10 min at 4°C and 12000 rpm, and the supernatant was used to measure enzymatic antioxidant activities and ROS. To analyze the H_2_O_2_ content, 0.1 ml of the above supernatant was mixed with phenol red solution and horseradish peroxidase. After a 10 min reaction, 0.2 mL of the supernatant was collected and mixed with 2 mL of 1 M NaOH before recording the absorbance of the supernatant at A_600_ and calculating the H_2_O_2_ content as previously reported [38].

Lipid peroxidation was determined by the thiobarbituric acid reactive substances assay (TBARS). Shoots and roots of Arabidopsis seedlings were extracted and centrifuged at 12,000 rpm for 10 min at 4°C. 2 mL of supernatant was mixed with 2 mL of 0.67% (w/v) thiobarbituric acid (TBA), boiled at 95°C for 30 min, and cooled immediately after centrifugation. The absorbance of the supernatant was measured at 532 nm and 600 nm using a UV/Vis spectrophotometer (UV-1801 UV-Vis spectrophotometer). The final malonyldialdehyde (MDA) concentration was calculated and expressed as previously reported [39]. Total protein was quantified by the Bradford method [40] with BSA as the standard. The activity of SOD, CAT, GSH, and GR was determined spectrophotometrically using detection kits (A001-3-2, A007-1-1, A006-2-1, A104-1-1 Jiancheng, Nanjing, China) according to the manufacturer’s instructions.

### Quantitative real-time PCR (qRT-PCR) analysis

The expression level of genes encoding antioxidant enzymes was investigated by qRT-PCR according to our previous study [41]. The roots and shoots of ten-day-old Arabidopsis seedlings were harvested separately and ground in liquid nitrogen to extract total RNA. Total RNA was extracted from the roots and shoots of ten-day-old Arabidopsis seedlings using the RNeasy Plant Mini Kit (Qiagen) according to the manufacturer’s instructions. The 1st strand of cDNA was reverse transcribed using SuperScript III Reverse Transcriptase (Invitrogen), and qRT-PCR analysis was performed using SYBR Premix Ex Taq II (Takara). The relative expression of target genes was calculated using the ΔΔCt method according to our previous study [41]. The constitutively expressed actin gene (*AtActin*) was used as an internal control. All primers used for qRT-PCR analysis were designed with primer 5.0 and are listed in S2 Table.

### Statistical analysis

In each experiment, more than 60 Arabidopsis seedlings of each treatment were used, and the experiment was repeated at least three times. All data obtained were statistically analyzed in SAS version 8.01 (SAS Institute Ltd., USA). Values shown are means ± standard errors (SE). Asterisks (*) indicate significant differences from control (* *P* < 0.05, ** *P* < 0.01, by Student’s *t*-test). Different letters indicate a significant difference between treatments (*P* < 0.05, by Duncan test).

## Results and discussion

### Effects of ZnO NPs and MWCNTs on the growth of Arabidopsis seedlings

In this study, we focused on the toxicity of ZnO NPs alone or in combination with WMCNT, so we did not make a comparison with the free Zn ions. However, according to the literature, insoluble ZnO NPs appear to be more toxic to many organisms than a similar amount of the ionic form of Zn^2+^ [42]. The underlying mechanism is that when treated with ZnO NPs, Zn^2+^ accumulated in the root where it competes with other metal ions and was therefore toxic to plants, whereas in the free Zn^2+^ treatment, due to its translocation from root to shoot, the negative effects were milder [11]. ZnO NPs at concentrations of 2000 and 400 mg/L have been reported to inhibit seed germination of Arabidopsis [10] and maize [17], respectively. In the present study, no obvious inhibition of Arabidopsis seed germination by ZnO NPs or MWCNTs was observed, which may be due to the much lower concentration we used and protection by the seed coat. Ten days after germination (10 DAG), phytotoxic signs were evident in Arabidopsis seedlings treated with ZnO NPs at concentrations of (30 and 50 mg/L), and root growth was more affected by the concentrations of ZnO NPs than by their size (Table 1). MWCNTs also showed phytotoxic effects on Arabidopsis with increasing size (10–20, 20–40 and 40–60 nm outer diameter) and concentration (50, 100, 200 and 500 mg/L) (Table 1).

**Table 1:** Root length and hypocotyls of Arabidopsis seedlings at 10 DAG after treatment with ZnO NPs and MWCNTs, respectively.

**ZnO NPs (mg/L)**	**Primary Root length (cm)**				**Hypocotyl Length (cm)**			
	**Φ90**		**Φ200**		**Φ90**		**Φ200**	
**0**	4.35±0.23^a^		4.35±0.23^a^		0.62±0.10^a^		0.62±0.10^a^	
**15**	4.70±0.30^a^		4.51±0.27^a^		0.65±0.13^a^		0.65±0.13^a^	
**30**	3.43±0.18^b^		3.10±0.11^b^		0.51±0.08^a^		0.51±0.08^a^	
**50**	1.10±0.19^c^		0.96±0.24^c^		0.36±0.08^b^		0.36±0.08^b^	
**MWCNTs (mg/L)**	**Primary Root length (cm)**				**Hypocotyl Length (cm)**			
	**Φ1020**	**Φ2040**		**Φ4060**	**Φ1020**	**Φ2040**		**Φ4060**
**0**	4.39±0.19^ab^	4.39±0.19^a^		4.39±0.19^a^	0.59±0.16^a^	0.59±0.16^a^		0.59±0.1^a^
**50**	4.72±0.28^a^	3.58±0.17^b^		3.30±0.33^b^	0.64±0.13^a^	0.64±0.22^ab^		0.86±0.2^b^
**100**	3.65±0.14^b^	3.05±0.19^bc^		2.67±0.21^c^	0.72±0.08^ab^	0.70±0.10^ab^		0.76±0.1^ab^
**200**	3.43±0.10^b^	2.85±0.10^bc^		2.48±0.18^c^	0.74±0.08^ab^	0.75±0.16^b^		0.75±0.2^ab^
**500**	2.9±0.17^c^	2.60±0.09^c^		2.60±0.09^c^	0.79±0.14^b^	0.74±0.03^b^		0.74±0.1^ab^

Previously, ZnO NPs at a concentration of 2000 mg/L were shown to reduce root growth of maize and terminate root development of five plant species, including radish, canola, ryegrass, lettuce, and cucumber; while MWCNTs showed no toxicity at 2000 mg/L in the same work [17]. In this study, ZnO NPs showed stronger inhibitory effect than MWCNTs (Table 1). Therefore, based on the concentrations that inhibit almost 50% of primary root growth, we used two concentrations (40 and 50 mg/L) for 90 nm diameter ZnO NPs and (100 and 500 mg/L) for 40–60 nm outer diameter MWCNTs in the following analysis.

**Fig 1:**
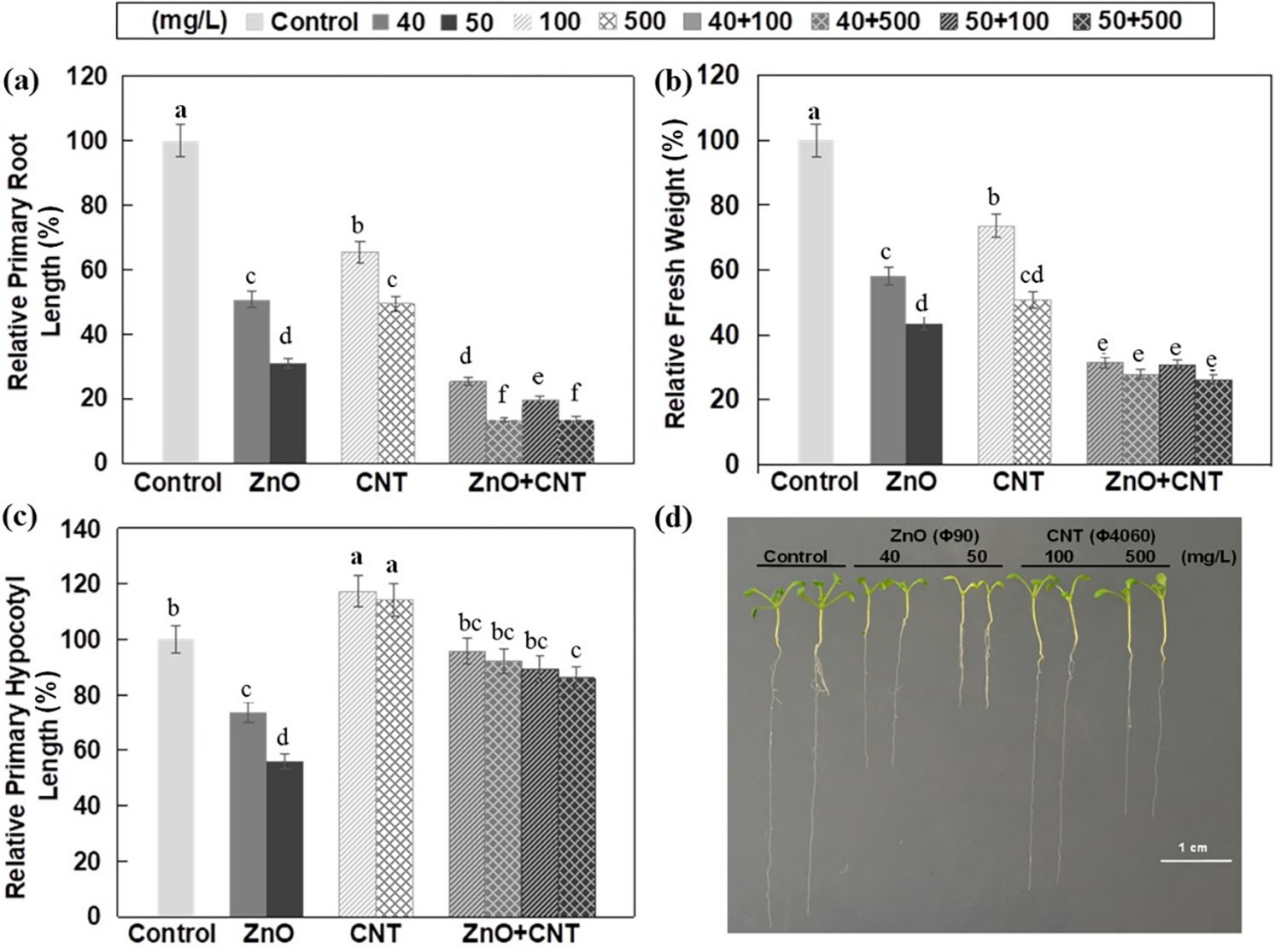
Effects of ZnO NPs and MWCNTs, alone or in combination, on Arabidopsis seedling growth at 10 DAG. (a) Relative primary root length. (b) Relative fresh weight. (c) Relative hypocotyl length. (d) Representative photographs of Arabidopsis seedlings. ½ MS Medium without NPs was used as control. Data are the means ± standard errors of three replicates. Different letters indicate a significant difference between treatments (*P* < 0.05, Duncan test).

When applied alone, ZnO NPs at a concentration of 40 and 50 mg/L caused a 49% and 69% reduction in primary root length, respectively, and MWCNTs at a concentration of 100 and 500 mg/L caused a 34% and 51% reduction in root length, respectively (Fig 1A). However, the combined effect of ZnO NPs and MWCNTs resulted in a 75%-86% reduction in primary root length, indicating an obvious additive effect (Fig 1A). Plant biomass is considered a sensitive biomarker of growth and development, and co-application of ZnO NPs and MWCNTs also showed an obvious synergistic effect on biomass (Fig 1B). Other than root length and fresh weight, MWCNTs and ZnO NPs moderately increased and decreased hypocotyl length, respectively, and when co-applied, the negative effect of ZnO NPs on hypocotyl elongation appeared to be mitigated by the presence of MWCNTs (Fig 1C).

### Effects of ZnO NPs and MWCNTs on the chlorophyll content in Arabidopsis seedlings

**Fig 2:**
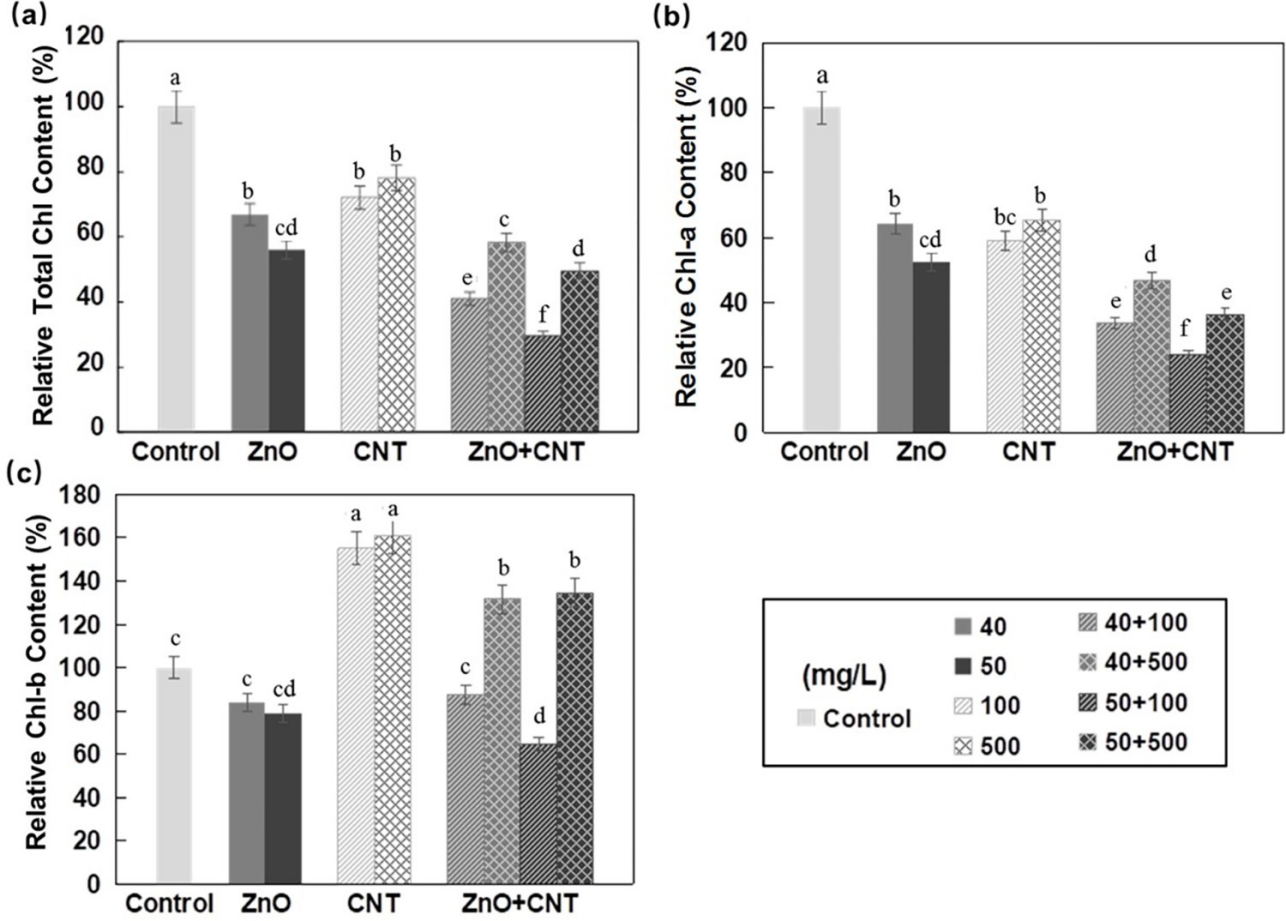
Effects of ZnO NPs and MWCNTs, alone or in combination, on chlorophyll content of Arabidopsis seedlings at 10 DAG. (a) Relative total chlorophyll content. (b) Relative chlorophyll-a content. (c) Relative chlorophyll-b content. ½ MS medium without NPs was used as control. Data are the means ± standard errors of three replicates. Different letters indicate a significant difference between treatments (*P* < 0.05, Duncan test).

When exposed to either ZnO NPs or MWCNTs, the total chlorophyll content of Arabidopsis seedlings was significantly decreased (Fig 2A). Although both decreased Chl-a content, treatment with ZnO NPs and MWCNTs decreased and increased Chl-b content, respectively (Fig 2B and 2C). When applied together, a synergistic effect was observed that further decreased total chlorophyll and Chl-a content. In the case of Chl-b, MWCNTs slightly reduced the decreasing effect of ZnO NPs (Fig 2C). Previously, a similar reduction in total chlorophyll content was reported in green leaves of *Pisum sativum*, *Salvinia natans*, and *Arabidopsis thaliana* treated with ZnO NPs [43–45], which was probably due to impaired photosynthesis and reduced biomass accumulation [46].

### Effects of ZnO NPs and MWCNTs on the level of MDA and H_2_O_2_ in Arabidopsis seedlings

**Fig 3:**
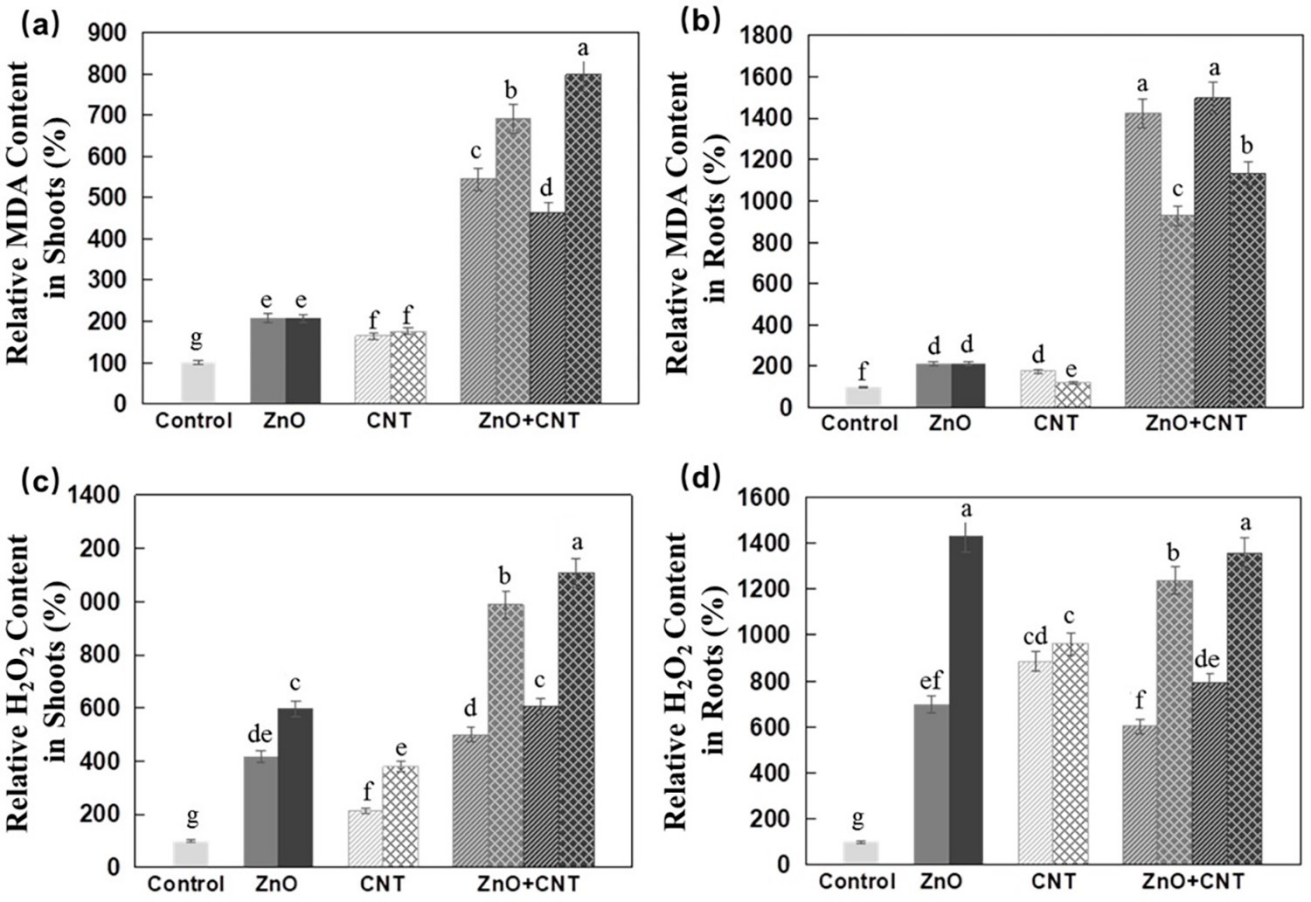
Effects of ZnO NPs and MWCNTs, alone or in combination, on MDA and H_2_O_2_ content of Arabidopsis seedlings at 10 DAG. (a) Relative MDA content in shoots. (b) Relative MDA content in roots. (c) Relative H_2_O_2_ content in shoots. (d) Relative H_2_O_2_ content in roots. ½ MS medium without NPs was used as control. Data are the means ± standard errors of three replicates. Different letters indicate a significant difference between treatments (*P* < 0.05, Duncan test).

In this study, significantly higher MDA levels were observed in Arabidopsis seedlings treated with either nanomaterial, with no apparent dose dependence, and an apparent synergistic effect was observed in the presence of both nanomaterials (Fig 3A and 3B). Both ZnO NPs and MWCNTs increased H_2_O_2_ levels in Arabidopsis shoots and roots, but the effect was more pronounced in roots (Fig 3C and 3D).

In shoots, the additive effect of both nanomaterials was much more dramatic at the higher concentration of MWCNTs. In roots, on the other hand, such an additive effect was not as obvious, and the overall effect of ZnO NPs plus MWCNTs led to a similar result as ZnO NPs alone (Fig 3C and 3D). One of the undesirable consequences of environmental stress is the extreme production of ROS [47], which leads to lipid peroxidation [48] and increased MDA level [49]. For example, higher MDA content was found in CuO NPs-stressed leaves of rice seedlings [49]. In another work, it was reported that MDA production in Arabidopsis seedlings was not affected by exposure to 250–500 mg/L CeO_2_ NPs or 25–2000 mg/L In_2_O_3_, but at 1000 mg/L CeO_2_, MDA formation was increased 2.5-fold [50] and similarly, increased ROS content was found in rice cells treated with 20 mg/L SWCNTs [21].

### Effects of ZnO NPs and MWCNTs on the activities of SOD, CAT and the expression of related genes

**Fig 4:**
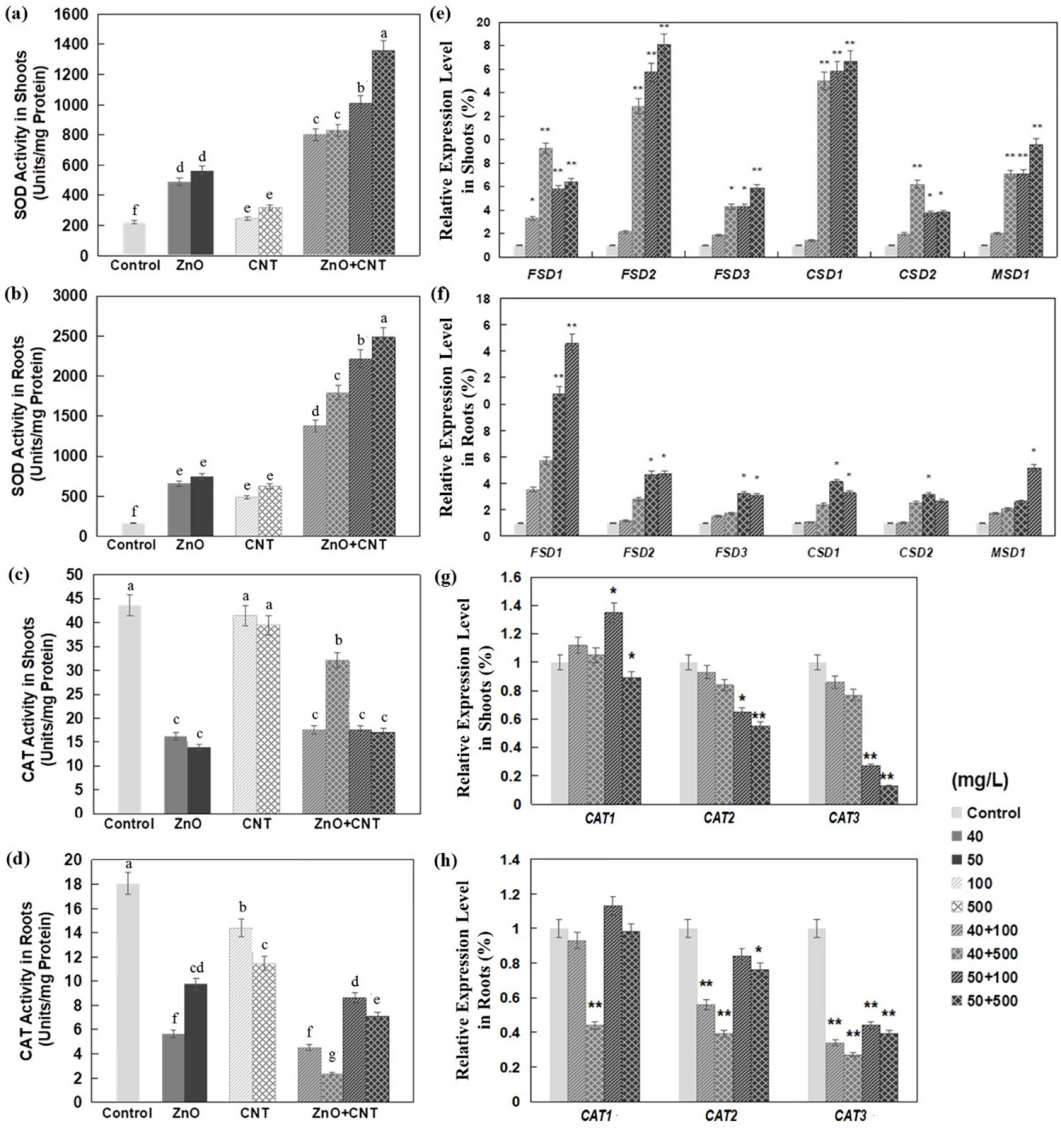
Effect of ZnO NPs and MWCNTs, alone or in combination, on enzyme activity and expression levels of genes encoding enzymes in Arabidopsis seedlings at 10 DAG. (a) SOD activity in shoots. (b) SOD activity in roots. (c) CAT activity in shoots. (d) CAT activity in roots. (e) Relative expression levels of genes encoding multiple SOD isoforms in shoots. (f) Relative expression levels of genes encoding multiple SOD isoforms in roots. (g) Relative expression levels of genes encoding multiple CAT isoforms in shoots. (h) Relative expression levels of genes encoding multiple CAT isoforms in roots. Level of gene expression in control seedlings was set to 1. Data are the means ± standard errors of three replicates. Different letters indicate a significant difference between treatments (*P* < 0.05, by Duncan test). *P<0.05 and **P<0.01 compared with control plants (by Student’s *t*-test).

In this study, total SOD activities were increased in Arabidopsis shoots and roots when exposed to either ZnO NPs or MWCNTs, especially in roots treated with ZnO NPs, with no significant dose effect (Fig 4A and 4B). SOD is a metalloenzyme that catalyzes the dismutation of superoxide radicals to O_2_ and H_2_O_2_, while CAT is another important enzyme involved in the antioxidant defense system by converting free radicals H_2_O_2_ to water and oxygen [51]. It was reported that the activities of SOD and CAT were increased 6.8- and 1.7-fold, respectively, in tomato plants treated with 2000 mg/L NiO NPs [52], suggesting that SOD and CAT could work together to decrease the total free radical content in cells.

When ZnO NPs and MWCNTs were used in combination, the total activity of SOD was increased in both shoots and roots with obvious synergistic effects (Fig 4A and 4B). It is known that the production of ROS occurs in different cellular compartments where different genes encoding enzymatic and non-enzymatic defenses are activated to protect the cell from oxidative influences [53]. In most higher plants, there are three classes of SOD enzymes [54] localized in different organelles, including copper/zinc SOD (Cu/Zn-SOD) in the cytosol, chloroplasts, and peroxisomes; iron SOD (Fe-SOD) in chloroplasts, peroxisomes, and apoplasts; and manganese SOD (Mn-SOD) in mitochondria [55]. Interestingly, the expression of Cu/Zn-SOD-coding genes, *CSD1* and *CSD2*, was repressed in a Cu-deficient medium and induced by the addition of Cu, whereas the expression of an Fe-SOD-coding gene, *FSD1*, was increased under Cu-deficient conditions and decreased in a Cu-containing medium [54]. This coordinated regulation of SOD-coding genes was also observed in the fern *Matteuccia struthiopteris* [55] and in higher plants such as tobacco [56] and Arabidopsis [57]. In this work, the transcriptional changes of SOD -encoding genes were studied in Arabidopsis seedlings simultaneously exposed to ZnO NPs and MWCNTs. It was found that *FSD1* was induced in roots and *FSD2*, *CSD1* followed by *MSD1*, and *FSD1* in shoots (Fig 4E and 4F). These results suggest that different gene regulatory patterns underline the increased SOD activities in different tissues. Significantly increased expression of *FSD*, *MSD*1, and *CSD1* genes was also detected in rice seedlings exposed to AgNPs [58].

In contrast to their induction of SOD activities, ZnO NPs and MWCNTs decreased CAT activities in Arabidopsis seedlings compared with control plants (Fig 4C and 4D). Literature reported that CAT activities of velvet mesquite and Brassica napus were significantly increased after exposure to 4000 mg/L ZnO NPs [59] and 500–4000 mg/L TiO_2_ NPs [60]. Similarly, 1000 mg/L CeO_2_ exposure increased the CAT activity of Arabidopsis by 3.5 to 4.0 times [20]. However, another report showed that CAT activity decreased by 50% in roots of rice cultivar Cheniere treated with 500 mg/L CeO_2_ NPs [61]. These studies indicated that the effects of exposure to NPs on the activity of CAT could depend on the type of NPs, concentration, and plant species. In the presence of both ZnO NPs and MWCNTs, the decrease in CAT activity appeared to be mainly dependent on the concentration of ZnO NPs (Fig 4C and 4D). Gene expression analysis showed that only *CAT3*, but not *CAT2* and *CAT1*, was significantly reduced in Arabidopsis shoots and roots (Fig 4G and 4H). This suggests that the reduced activity of CAT is mainly due to the reduced expression of *CAT3*, whose gene product is presumably localized in the mitochondria.

### Effects of ZnO NPs and MWCNTs on GSH level, GR activity and related gene expression

**Fig 5:**
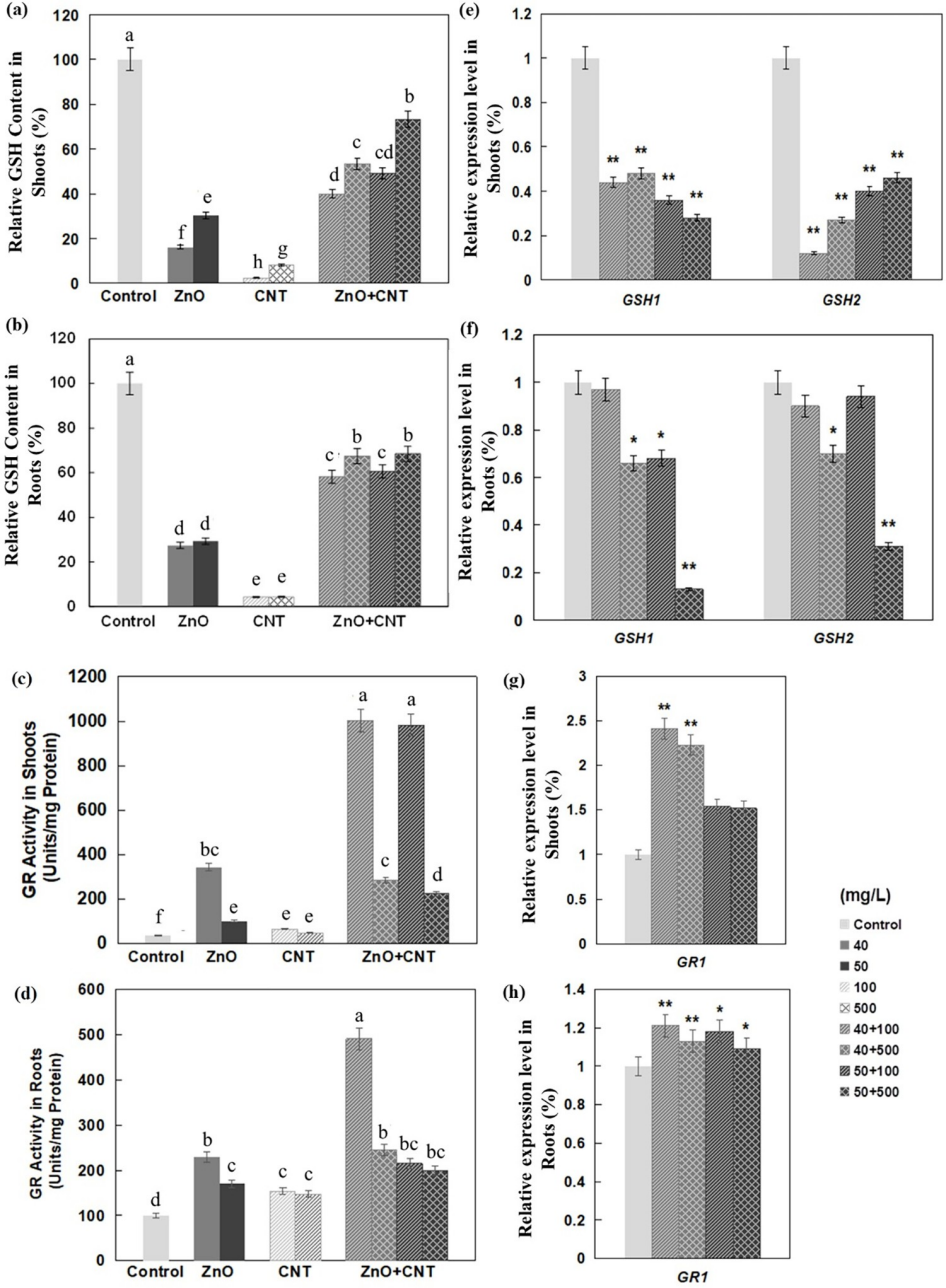
Effect of ZnO NPs and MWCNTs, alone or in combination, on GSH content, GR activities, and expression levels of genes encoding GSH, GR isoforms in Arabidopsis seedlings at 10 DAG. (a) Relative GSH content in shoots. (b) Relative GSH content in roots. (c) GR activity in shoots. (d) GR activity in roots. (e) Relative expression levels of genes encoding multiple GSH isoforms in shoots. (f) Relative expression levels of genes encoding multiple GSH isoforms in roots. (g) Relative expression levels of genes encoding multiple GR isoforms in shoots. (h) Relative expression levels of genes encoding multiple GR isoforms in roots. Data are the means ± standard errors of three replicates. Different letters indicate a significant difference between treatments (*P* < 0.05, by Duncan test). *P<0.05 and **P<0.01 compared with control plants (by Student’s *t*-test).

In this work, both ZnO NPs and MWCNTs drastically reduced GSH content in Arabidopsis shoots and roots, with a more negative effect for MWCNTs (Fig 5A and 5B). Interestingly, the reduction in GSH content in shoots and roots was much lower when both nanomaterials were applied together (Fig 5A and 5B), which was accompanied by reduced expression of *GSH1* and *GSH2* (Fig 5E and 5F). In contrast, significant up-regulation of genes involved in both sulfur assimilation and GSH biosynthetic pathways was induced by exposure to CeO_2_ or In_2_O_3_ NPs in Arabidopsis [50]. GSH is mainly localized in chloroplasts and is one of the most important antioxidant molecules in cells [62]. GSH can directly degrade H_2_O_2_ in the ascorbate-glutathione cycle and its level is considered a sensitive indicator of oxidative stress in plants [63]. The glutathione synthesis pathway in plants involves two ATP-dependent enzymes, γ-glutamylcysteine synthetase (GSH1) and glutathione synthetase (GSH2), which are encoded by single genes (*GSH1* and *GSH2*, respectively) in Arabidopsis [64].

By reducing GSSG to GSH, higher GR activity can improve the efficiency of GSH-mediated detoxification [63]. In another report, it was shown that GR activity was increased in the roots of rice cultivar (Neptune) when exposed to 62.5 and 500 mg/L CeO_2_ NPs, while it decreased at the other two concentrations (125, 250 mg/L) [61]. In the present study, both ZnO NPs and MWCNTs increased GR activities, and a remarkable synergistic effect was observed when they were applied in combination (Fig 5C and 5D). *GR1* mainly encodes a cytosolic glutathione reductase [65] and simultaneous exposure to two nanomaterials resulted in significant upregulation of *GR1* only in shoots but not in roots (Fig 5G and 5H), suggesting that the increased GR activities in these two tissues may be due to different molecular mechanisms.

## Conclusion

The present study aimed to understand the phytotoxic effects of two nanomaterials, ZnO NPs and MWCNTs, when applied alone or simultaneously to seedlings of the model plant *Arabidopsis thaliana*. When applied alone, ZnO NPs caused stronger inhibitory effects than MWCNTs on several plant growth indices, including reduced root length, chlorophyll content, and increased ROS concentration. When applied simultaneously, the combined effects of both nanomaterials were generally more toxic than those induced by ZnO NPs or MWCNTs. However, while most growth parameters of Arabidopsis seedlings were negatively affected in a synergistic or additive manner, GSH content was significantly decreased in the presence of both nanomaterials, suggesting an antagonistic effect. Moreover, the negative effects of ZnO NPs on hypocotyl elongation and Chl-b content appeared to be attenuated by MWCNTs. Moreover, this work showed that Arabidopsis seedlings simultaneously exposed to two nanomaterials regulated the expression of distinctive sets of genes involved in antioxidant defense to cope with ROS stress.

## Supporting information

S1 Table The information of ZnO NPs and MWCNTs provided by the manufacturer. (PDF)

S2 Table Primers used for qRT-PCR analysis. (PDF)
