# Supplementary material for: Phytotoxicity of zinc oxide nanoparticles and multi-walled carbon nanotubes, alone or in combination, on Arabidopsis thaliana and their mutual effects on oxidative homeostasis
Source: PLoS One. 2023 Feb 15;18(2):e0281756. doi: 10.1371/journal.pone.0281756 (PMC9931106; doi:10.1371/journal.pone.0281756)
Supplement: S1 Table — (PDF) [file pone.0281756.s001.pdf]

S1 Table. The information of ZnO NPs and MWCNTs provided by the manufacturer.

Nanomaterials		Particle size (nm)	Purity (%)	Density (g/cm^3)	Special surface area (m^2/g)	Manufacturer	CAS number /Model
**ZnO**	Φ90	90 ± 10	＞99.8	5.61	15-25	Shanghai Macklin Biochemical Co., Ltd (Shanghai, China)	Z820773
	Φ200	200	＞99.9	5.61	---		Z820825
**MWCNTs**	Φ1020	OD: 10-20<br>Length: ＜2 μm	＞97	0.27-0.40	100-160	Shenzhen Nanotech Port Co. Ltd (Shenzhen, China)	S-MWNT-1020
	Φ2040	OD: 20-40<br>Length: ＜2 μm	＞97	0.03-0.16	70-150		S-MWNT-2040
	Φ4060	OD: 40-60<br>Length: ＜2 μm	＞97	0.03-016	60-160		S-MWNT-4060
