# Supplementary material for: Phytotoxicity of zinc oxide nanoparticles and multi-walled carbon nanotubes, alone or in combination, on Arabidopsis thaliana and their mutual effects on oxidative homeostasis
Source: PLoS One. 2023 Feb 15;18(2):e0281756. doi: 10.1371/journal.pone.0281756 (PMC9931106; doi:10.1371/journal.pone.0281756)
Supplement: S2 Table — (PDF) [file pone.0281756.s002.pdf]

S2 Table. Primers used for qRT-PCR analysis.

Genes	Primer Sequences for qRT-PCR（5'—3'）	
	F	R
AtFSD1	CTCAAGCCACCTCCATTCG	GCGTTGTTGAAAGCAGGGA
AtFSD2	TGGATTATCACTGGGGCAAAC	GGATAGACTCCCAGAAGAACTCG
AtFSD3	GTGAACCCAACATCCCAATCG	TTGCGTCACTAACATTACTGTCACC
AtCSD1	AGTAACCAAAGAGAGACGAAGCA	CCTTCCTGGGTGAAAAAGATAG
AtCSD2	CGAAGGAGTTGTTACTTTGACCC	GAACCACAAAGGCTCTTCCAAC
AtMSD1	GTTTGGGAGCACGCCTACTAC	GTTCATCTCCTTATGTCATCGTGTA
AtCAT1	CGCCGATTTGCGAGATACA	ACCCTCTCAGGAATCCGCTC
AtCAT2	TATCCAACTCCGCCTGCTGTCT	ATGCGTGGGTCGGATAGGG
AtCAT3	GGTGACACTCAGAGACATCGCC	AAACCTGTCTTGCCTGTCTGG
AtGSH1	TTTGAGCAGTATGTTGACTACGCAC	GCAGTTCACCAGGGAGACAGG
AtGSH2	GGAAATGCTTTGCTGGGC	TCTCCATAGATGTTGTTTCCTCC
At-Actin	CAGTGGTGGTGAATGAGTAGCC	CAAAAGCAAACAGAGAAAAGATGA
